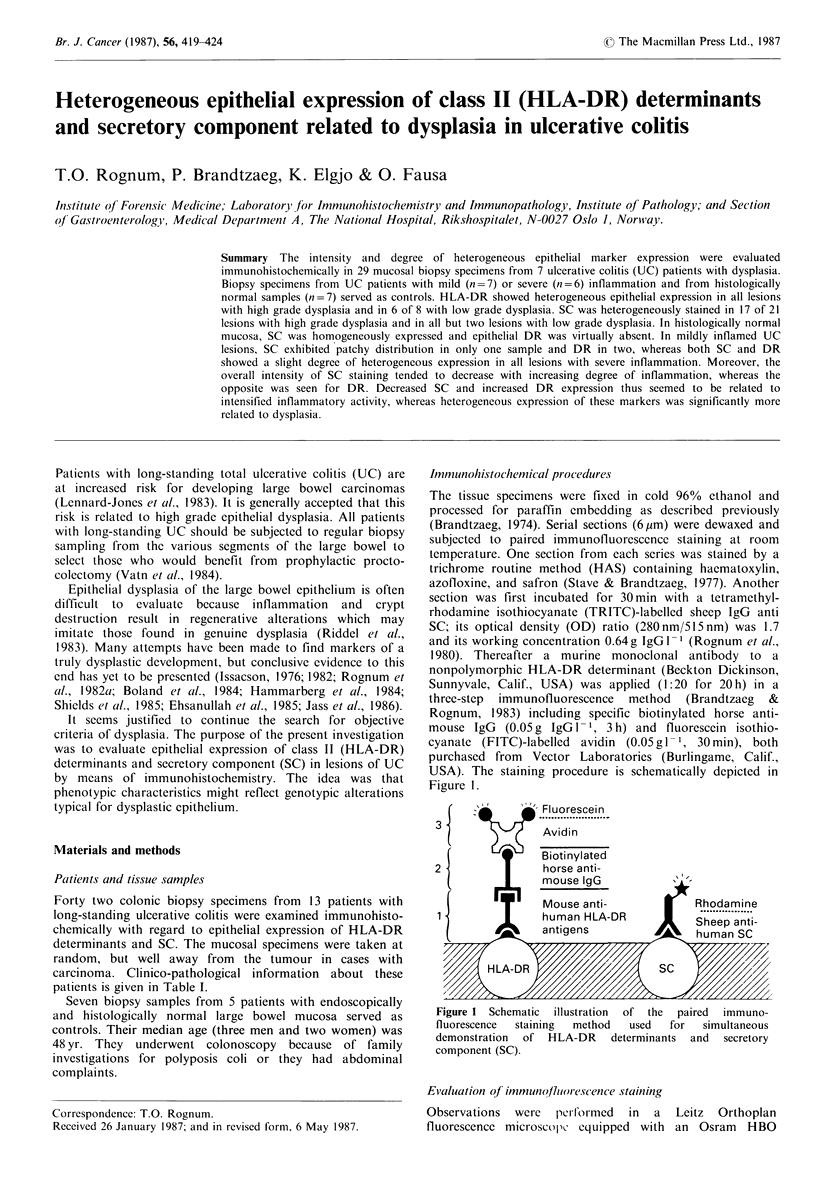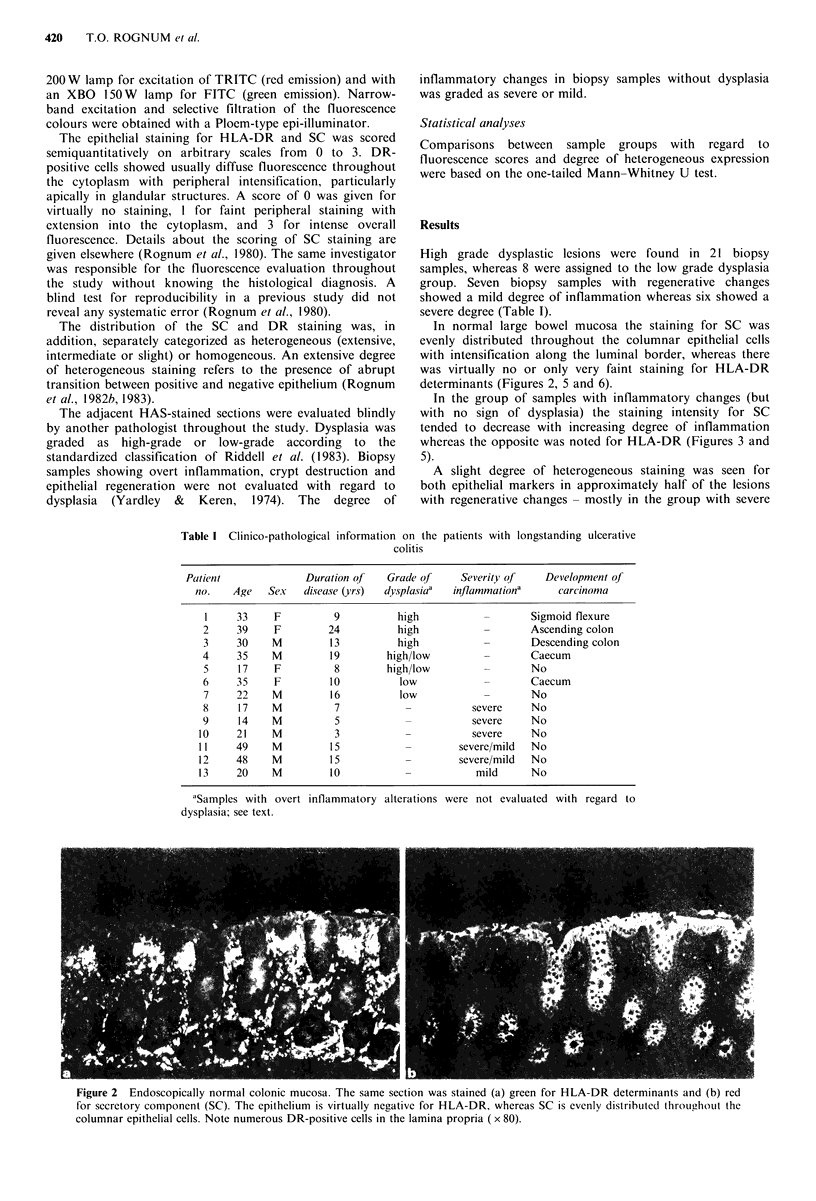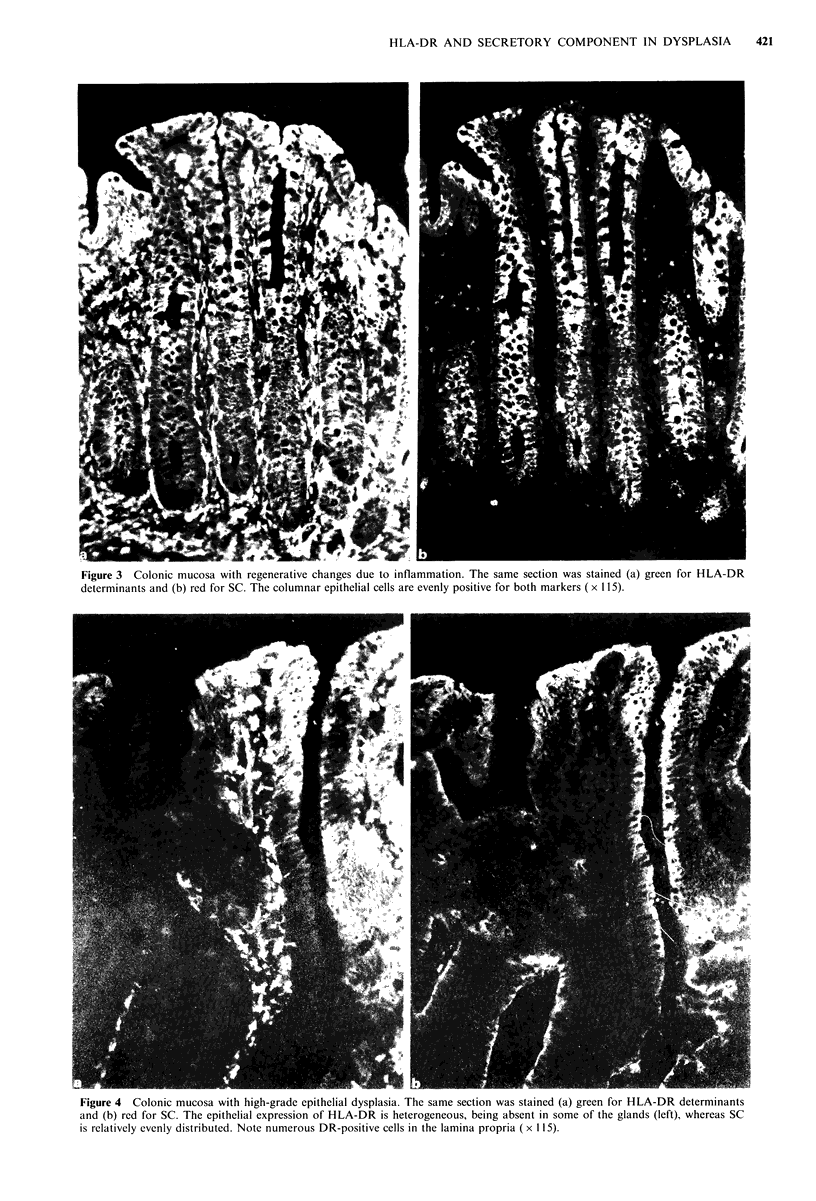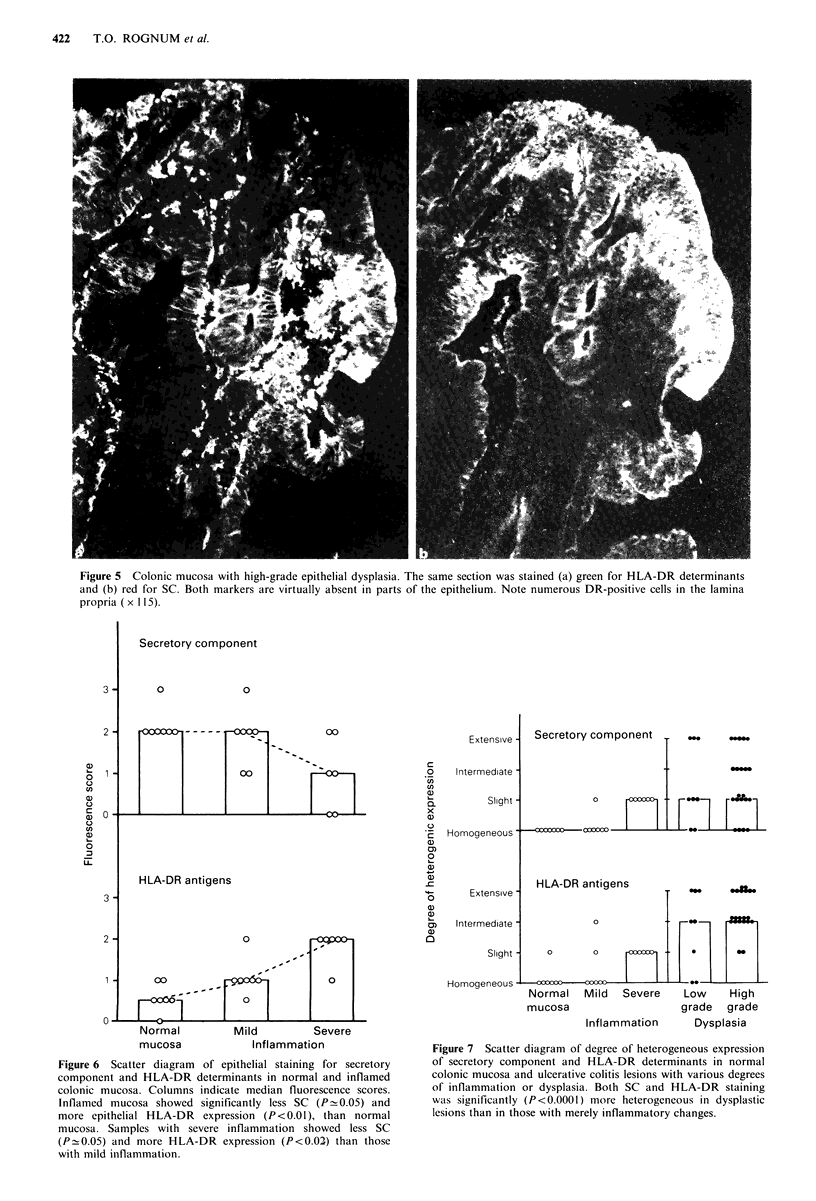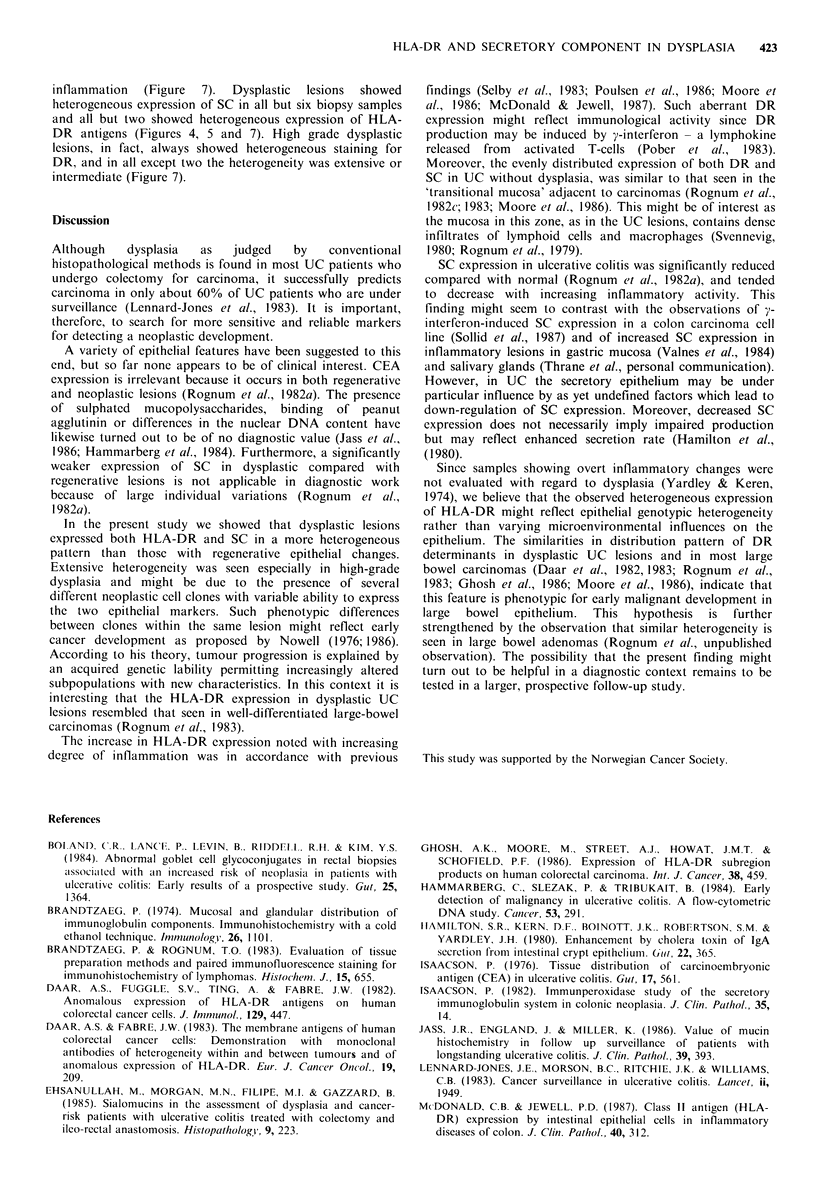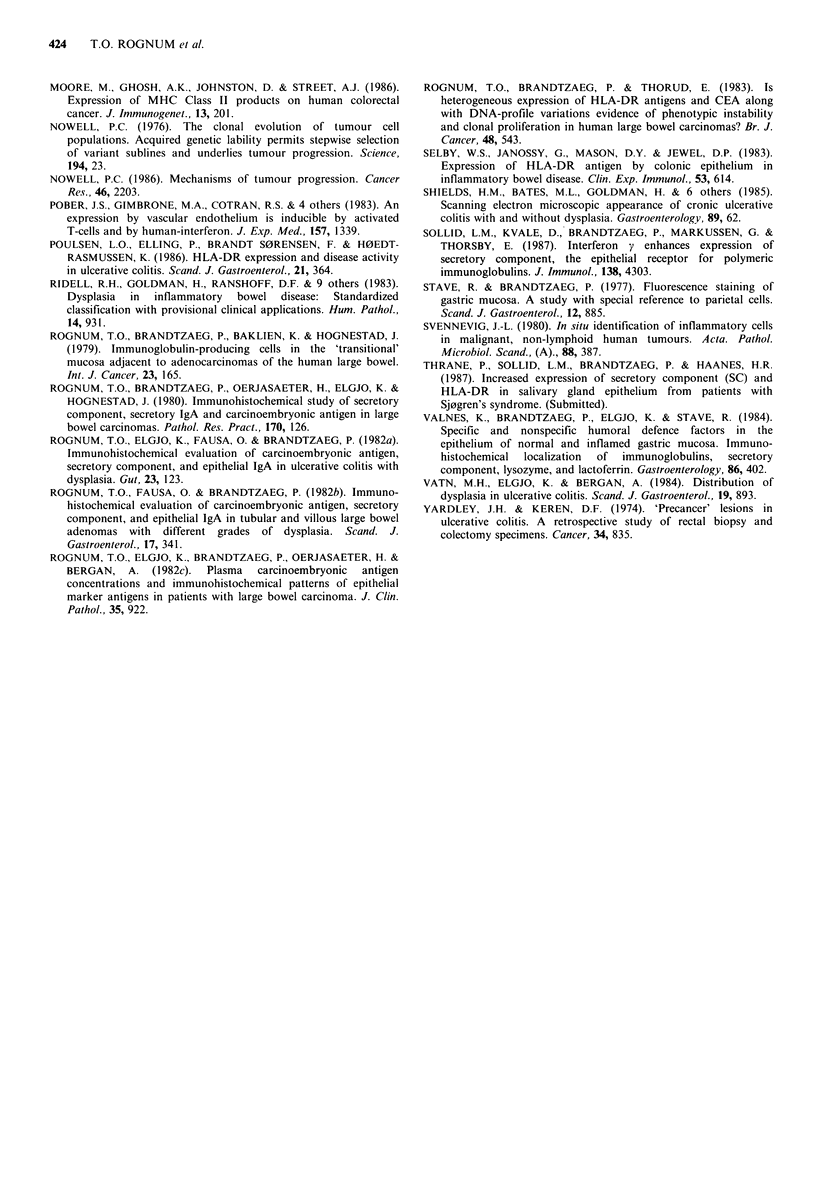# Heterogeneous epithelial expression of class II (HLA-DR) determinants and secretory component related to dysplasia in ulcerative colitis.

**DOI:** 10.1038/bjc.1987.217

**Published:** 1987-10

**Authors:** T. O. Rognum, P. Brandtzaeg, K. Elgjo, O. Fausa

**Affiliations:** Institute of Forensic Medicine, Oslo, Norway.

## Abstract

**Images:**


*Br. J. Cancer* (1987), **56**, 419–424 © The Macmillan Press Ltd., 1987

### Heterogeneous epithelial expression of class II (HLA-DR) determinants and secretory component related to dysplasia in ulcerative colitis

T.O. Rognum, P. Brandtzaeg, K. Elgjo & O. Fausa

*Institute of Forensic Medicine; Laboratory for Immunohistochemistry and Immunopathology, Institute of Pathology; and Section of Gastroenterology, Medical Department A, The National Hospital, Rikshospitalet, N-0027 Oslo 1, Norway.*

**Summary** The intensity and degree of heterogeneous epithelial marker expression were evaluated immunohistochemically in 29 mucosal biopsy specimens from 7 ulcerative colitis (UC) patients with dysplasia. Biopsy specimens from UC patients with mild ($n=7$) or severe ($n=6$) inflammation and from histologically normal samples ($n=7$) served as controls. HLA-DR showed heterogeneous epithelial expression in all lesions with high grade dysplasia and in 6 of 8 with low grade dysplasia. SC was heterogeneously stained in 17 of 21 lesions with high grade dysplasia and in all but two lesions with low grade dysplasia. In histologically normal mucosa, SC was homogeneously expressed and epithelial DR was virtually absent. In mildly inflamed UC lesions, SC exhibited patchy distribution in only one sample and DR in two, whereas both SC and DR showed a slight degree of heterogeneous expression in all lesions with severe inflammation. Moreover, the overall intensity of SC staining tended to decrease with increasing degree of inflammation, whereas the opposite was seen for DR. Decreased SC and increased DR expression thus seemed to be related to intensified inflammatory activity, whereas heterogeneous expression of these markers was significantly more related to dysplasia.

Patients with long-standing total ulcerative colitis (UC) are at increased risk for developing large bowel carcinomas (Lennard-Jones *et al.*, 1983). It is generally accepted that this risk is related to high grade epithelial dysplasia. All patients with long-standing UC should be subjected to regular biopsy sampling from the various segments of the large bowel to select those who would benefit from prophylactic procto-colectomy (Vatn *et al.*, 1984).

Epithelial dysplasia of the large bowel epithelium is often difficult to evaluate because inflammation and crypt destruction result in regenerative alterations which may imitate those found in genuine dysplasia (Riddel *et al.*, 1983). Many attempts have been made to find markers of a truly dysplastic development, but conclusive evidence to this end has yet to be presented (Issacson, 1976; 1982; Rognum *et al.*, 1982*a*; Boland *et al.*, 1984; Hammarberg *et al.*, 1984; Shields *et al.*, 1985; Ehsanullah *et al.*, 1985; Jass *et al.*, 1986).

It seems justified to continue the search for objective criteria of dysplasia. The purpose of the present investigation was to evaluate epithelial expression of class II (HLA-DR) determinants and secretory component (SC) in lesions of UC by means of immunohistochemistry. The idea was that phenotypic characteristics might reflect genotypic alterations typical for dysplastic epithelium.

### Materials and methods

### *Patients and tissue samples*

Forty two colonic biopsy specimens from 13 patients with long-standing ulcerative colitis were examined immunohisto-chemically with regard to epithelial expression of HLA-DR determinants and SC. The mucosal specimens were taken at random, but well away from the tumour in cases with carcinoma. Clinico-pathological information about these patients is given in Table I.

Seven biopsy samples from 5 patients with endoscopically and histologically normal large bowel mucosa served as controls. Their median age (three men and two women) was 48 yr. They underwent colonoscopy because of family investigations for polyposis coli or they had abdominal complaints.

Correspondence: T.O. Rognum.
Received 26 January 1987; and in revised form, 6 May 1987.

### *Immunohistochemical procedures*

The tissue specimens were fixed in cold 96% ethanol and processed for paraffin embedding as described previously (Brandtzaeg, 1974). Serial sections (6 $\mu$m) were dewaxed and subjected to paired immunofluorescence staining at room temperature. One section from each series was stained by a trichrome routine method (HAS) containing haematoxylin, azofloxine, and safron (Stave & Brandtzaeg, 1977). Another section was first incubated for 30 min with a tetramethyl-rhodamine isothiocyanate (TRITC)-labelled sheep IgG anti SC; its optical density (OD) ratio (280 nm/515 nm) was 1.7 and its working concentration 0.64 g IgG $l^{-1}$ (Rognum *et al.*, 1980). Thereafter a murine monoclonal antibody to a nonpolymorphic HLA-DR determinant (Beckton Dickinson, Sunnyvale, Calif., USA) was applied (1:20 for 20 h) in a three-step immunofluorescence method (Brandtzaeg & Rognum, 1983) including specific biotinylated horse anti-mouse IgG (0.05 g IgG $l^{-1}$, 3 h) and fluorescein isothio-cyanate (FITC)-labelled avidin (0.05 g $l^{-1}$, 30 min), both purchased from Vector Laboratories (Burlingame, Calif., USA). The staining procedure is schematically depicted in Figure 1.

**Figure 1** Schematic illustration of the paired immuno-fluorescence staining method used for simultaneous demonstration of HLA-DR determinants and secretory component (SC).

### *Evaluation of immunofluorescence staining*

Observations were performed in a Leitz Orthoplan fluorescence microscope equipped with an Osram HBO

200 W lamp for excitation of TRITC (red emission) and with an XBO 150 W lamp for FITC (green emission). Narrow-band excitation and selective filtration of the fluorescence colours were obtained with a Ploem-type epi-illuminator.

The epithelial staining for HLA-DR and SC was scored semiquantitatively on arbitrary scales from 0 to 3. DR-positive cells showed usually diffuse fluorescence throughout the cytoplasm with peripheral intensification, particularly apically in glandular structures. A score of 0 was given for virtually no staining, 1 for faint peripheral staining with extension into the cytoplasm, and 3 for intense overall fluorescence. Details about the scoring of SC staining are given elsewhere (Rognum *et al.*, 1980). The same investigator was responsible for the fluorescence evaluation throughout the study without knowing the histological diagnosis. A blind test for reproducibility in a previous study did not reveal any systematic error (Rognum *et al.*, 1980).

The distribution of the SC and DR staining was, in addition, separately categorized as heterogeneous (extensive, intermediate or slight) or homogeneous. An extensive degree of heterogeneous staining refers to the presence of abrupt transition between positive and negative epithelium (Rognum *et al.*, 1982*b*, 1983).

The adjacent HAS-stained sections were evaluated blindly by another pathologist throughout the study. Dysplasia was graded as high-grade or low-grade according to the standardized classification of Riddell *et al.* (1983). Biopsy samples showing overt inflammation, crypt destruction and epithelial regeneration were not evaluated with regard to dysplasia (Yardley & Keren, 1974). The degree of inflammatory changes in biopsy samples without dysplasia was graded as severe or mild.

*Statistical analyses*

Comparisons between sample groups with regard to fluorescence scores and degree of heterogeneous expression were based on the one-tailed Mann–Whitney U test.

### Results

High grade dysplastic lesions were found in 21 biopsy samples, whereas 8 were assigned to the low grade dysplasia group. Seven biopsy samples with regenerative changes showed a mild degree of inflammation whereas six showed a severe degree (Table I).

In normal large bowel mucosa the staining for SC was evenly distributed throughout the columnar epithelial cells with intensification along the luminal border, whereas there was virtually no or only very faint staining for HLA-DR determinants (Figures 2, 5 and 6).

In the group of samples with inflammatory changes (but with no sign of dysplasia) the staining intensity for SC tended to decrease with increasing degree of inflammation whereas the opposite was noted for HLA-DR (Figures 3 and 5).

A slight degree of heterogeneous staining was seen for both epithelial markers in approximately half of the lesions with regenerative changes – mostly in the group with severe

**Table I** Clinico-pathological information on the patients with longstanding ulcerative colitis

| *Patient no.* | *Age* | *Sex* | *Duration of disease (yrs)* | *Grade of dysplasia*[a] | *Severity of inflammation*[a] | *Development of carcinoma* |
|---|---|---|---|---|---|---|
| 1 | 33 | F | 9 | high | – | Sigmoid flexure |
| 2 | 39 | F | 24 | high | – | Ascending colon |
| 3 | 30 | M | 13 | high | – | Descending colon |
| 4 | 35 | M | 19 | high/low | – | Caecum |
| 5 | 17 | F | 8 | high/low | – | No |
| 6 | 35 | F | 10 | low | – | Caecum |
| 7 | 22 | M | 16 | low | – | No |
| 8 | 17 | M | 7 | – | severe | No |
| 9 | 14 | M | 5 | – | severe | No |
| 10 | 21 | M | 3 | – | severe | No |
| 11 | 49 | M | 15 | – | severe/mild | No |
| 12 | 48 | M | 15 | – | severe/mild | No |
| 13 | 20 | M | 10 | – | mild | No |

[a]Samples with overt inflammatory alterations were not evaluated with regard to dysplasia; see text.

**Figure 2** Endoscopically normal colonic mucosa. The same section was stained (a) green for HLA-DR determinants and (b) red for secretory component (SC). The epithelium is virtually negative for HLA-DR, whereas SC is evenly distributed throughout the columnar epithelial cells. Note numerous DR-positive cells in the lamina propria (×80).

**Figure 3** Colonic mucosa with regenerative changes due to inflammation. The same section was stained (a) green for HLA-DR determinants and (b) red for SC. The columnar epithelial cells are evenly positive for both markers (×115).

**Figure 4** Colonic mucosa with high-grade epithelial dysplasia. The same section was stained (a) green for HLA-DR determinants and (b) red for SC. The epithelial expression of HLA-DR is heterogeneous, being absent in some of the glands (left), whereas SC is relatively evenly distributed. Note numerous DR-positive cells in the lamina propria (×115).

**Figure 5** Colonic mucosa with high-grade epithelial dysplasia. The same section was stained (a) green for HLA-DR determinants and (b) red for SC. Both markers are virtually absent in parts of the epithelium. Note numerous DR-positive cells in the lamina propria (× 115).

**Figure 6** Scatter diagram of epithelial staining for secretory component and HLA-DR determinants in normal and inflamed colonic mucosa. Columns indicate median fluorescence scores. Inflamed mucosa showed significantly less SC ($P \simeq 0.05$) and more epithelial HLA-DR expression ($P < 0.01$), than normal mucosa. Samples with severe inflammation showed less SC ($P \simeq 0.05$) and more HLA-DR expression ($P < 0.02$) than those with mild inflammation.

**Figure 7** Scatter diagram of degree of heterogeneous expression of secretory component and HLA-DR determinants in normal colonic mucosa and ulcerative colitis lesions with various degrees of inflammation or dysplasia. Both SC and HLA-DR staining was significantly ($P < 0.0001$) more heterogeneous in dysplastic lesions than in those with merely inflammatory changes.

inflammation (Figure 7). Dysplastic lesions showed heterogeneous expression of SC in all but six biopsy samples and all but two showed heterogeneous expression of HLA-DR antigens (Figures 4, 5 and 7). High grade dysplastic lesions, in fact, always showed heterogeneous staining for DR, and in all except two the heterogeneity was extensive or intermediate (Figure 7).

### Discussion

Although dysplasia as judged by conventional histopathological methods is found in most UC patients who undergo colectomy for carcinoma, it successfully predicts carcinoma in only about 60% of UC patients who are under surveillance (Lennard-Jones *et al.*, 1983). It is important, therefore, to search for more sensitive and reliable markers for detecting a neoplastic development.

A variety of epithelial features have been suggested to this end, but so far none appears to be of clinical interest. CEA expression is irrelevant because it occurs in both regenerative and neoplastic lesions (Rognum *et al.*, 1982*a*). The presence of sulphated mucopolysaccharides, binding of peanut agglutinin or differences in the nuclear DNA content have likewise turned out to be of no diagnostic value (Jass *et al.*, 1986; Hammarberg *et al.*, 1984). Furthermore, a significantly weaker expression of SC in dysplastic compared with regenerative lesions is not applicable in diagnostic work because of large individual variations (Rognum *et al.*, 1982*a*).

In the present study we showed that dysplastic lesions expressed both HLA-DR and SC in a more heterogeneous pattern than those with regenerative epithelial changes. Extensive heterogeneity was seen especially in high-grade dysplasia and might be due to the presence of several different neoplastic cell clones with variable ability to express the two epithelial markers. Such phenotypic differences between clones within the same lesion might reflect early cancer development as proposed by Nowell (1976; 1986). According to his theory, tumour progression is explained by an acquired genetic lability permitting increasingly altered subpopulations with new characteristics. In this context it is interesting that the HLA-DR expression in dysplastic UC lesions resembled that seen in well-differentiated large-bowel carcinomas (Rognum *et al.*, 1983).

The increase in HLA-DR expression noted with increasing degree of inflammation was in accordance with previous findings (Selby *et al.*, 1983; Poulsen *et al.*, 1986; Moore *et al.*, 1986; McDonald & Jewell, 1987). Such aberrant DR expression might reflect immunological activity since DR production may be induced by $\gamma$-interferon – a lymphokine released from activated T-cells (Pober *et al.*, 1983). Moreover, the evenly distributed expression of both DR and SC in UC without dysplasia, was similar to that seen in the 'transitional mucosa' adjacent to carcinomas (Rognum *et al.*, 1982*c*; 1983; Moore *et al.*, 1986). This might be of interest as the mucosa in this zone, as in the UC lesions, contains dense infiltrates of lymphoid cells and macrophages (Svennevig, 1980; Rognum *et al.*, 1979).

SC expression in ulcerative colitis was significantly reduced compared with normal (Rognum *et al.*, 1982*a*), and tended to decrease with increasing inflammatory activity. This finding might seem to contrast with the observations of $\gamma$-interferon-induced SC expression in a colon carcinoma cell line (Sollid *et al.*, 1987) and of increased SC expression in inflammatory lesions in gastric mucosa (Valnes *et al.*, 1984) and salivary glands (Thrane *et al.*, personal communication). However, in UC the secretory epithelium may be under particular influence by as yet undefined factors which lead to down-regulation of SC expression. Moreover, decreased SC expression does not necessarily imply impaired production but may reflect enhanced secretion rate (Hamilton *et al.*, (1980).

Since samples showing overt inflammatory changes were not evaluated with regard to dysplasia (Yardley & Keren, 1974), we believe that the observed heterogeneous expression of HLA-DR might reflect epithelial genotypic heterogeneity rather than varying microenvironmental influences on the epithelium. The similarities in distribution pattern of DR determinants in dysplastic UC lesions and in most large bowel carcinomas (Daar *et al.*, 1982, 1983; Rognum *et al.*, 1983; Ghosh *et al.*, 1986; Moore *et al.*, 1986), indicate that this feature is phenotypic for early malignant development in large bowel epithelium. This hypothesis is further strengthened by the observation that similar heterogeneity is seen in large bowel adenomas (Rognum *et al.*, unpublished observation). The possibility that the present finding might turn out to be helpful in a diagnostic context remains to be tested in a larger, prospective follow-up study.

This study was supported by the Norwegian Cancer Society.

### References

BOLAND, C.R., LANCE, P., LEVIN, B., RIDDELL, R.H. & KIM, Y.S. (1984). Abnormal goblet cell glycoconjugates in rectal biopsies associated with an increased risk of neoplasia in patients with ulcerative colitis: Early results of a prospective study. *Gut*, **25**, 1364.

BRANDTZAEG, P. (1974). Mucosal and glandular distribution of immunoglobulin components. Immunohistochemistry with a cold ethanol technique. *Immunology*, **26**, 1101.

BRANDTZAEG, P. & ROGNUM, T.O. (1983). Evaluation of tissue preparation methods and paired immunofluorescence staining for immunohistochemistry of lymphomas. *Histochem. J.*, **15**, 655.

DAAR, A.S., FUGGLE, S.V., TING, A. & FABRE, J.W. (1982). Anomalous expression of HLA-DR antigens on human colorectal cancer cells. *J. Immunol.*, **129**, 447.

DAAR, A.S. & FABRE, J.W. (1983). The membrane antigens of human colorectal cancer cells: Demonstration with monoclonal antibodies of heterogeneity within and between tumours and of anomalous expression of HLA-DR. *Eur. J. Cancer Oncol.*, **19**, 209.

EHSANULLAH, M., MORGAN, M.N., FILIPE, M.I. & GAZZARD, B. (1985). Sialomucins in the assessment of dysplasia and cancer-risk patients with ulcerative colitis treated with colectomy and ileo-rectal anastomosis. *Histopathology*, **9**, 223.

GHOSH, A.K., MOORE, M., STREET, A.J., HOWAT, J.M.T. & SCHOFIELD, P.F. (1986). Expression of HLA-DR subregion products on human colorectal carcinoma. *Int. J. Cancer*, **38**, 459.

HAMMARBERG, C., SLEZAK, P. & TRIBUKAIT, B. (1984). Early detection of malignancy in ulcerative colitis. A flow-cytometric DNA study. *Cancer*, **53**, 291.

HAMILTON, S.R., KERN, D.F., BOINOTT, J.K., ROBERTSON, S.M. & YARDLEY, J.H. (1980). Enhancement by cholera toxin of IgA secretion from intestinal crypt epithelium. *Gut*, **22**, 365.

ISAACSON, P. (1976). Tissue distribution of carcinoembryonic antigen (CEA) in ulcerative colitis. *Gut*, **17**, 561.

ISAACSON, P. (1982). Immunperoxidase study of the secretory immunoglobulin system in colonic neoplasia. *J. Clin. Pathol.*, **35**, 14.

JASS, J.R., ENGLAND, J. & MILLER, K. (1986). Value of mucin histochemistry in follow up surveillance of patients with longstanding ulcerative colitis. *J. Clin. Pathol.*, **39**, 393.

LENNARD-JONES, J.E., MORSON, B.C., RITCHIE, J.K. & WILLIAMS, C.B. (1983). Cancer surveillance in ulcerative colitis. *Lancet*, **ii**, 1949.

McDONALD, C.B. & JEWELL, P.D. (1987). Class II antigen (HLA-DR) expression by intestinal epithelial cells in inflammatory diseases of colon. *J. Clin. Pathol.*, **40**, 312.

MOORE, M., GHOSH, A.K., JOHNSTON, D. & STREET, A.J. (1986). Expression of MHC Class II products on human colorectal cancer. *J. Immunogenet.*, **13**, 201.

NOWELL, P.C. (1976). The clonal evolution of tumour cell populations. Acquired genetic lability permits stepwise selection of variant sublines and underlies tumour progression. *Science*, **194**, 23.

NOWELL, P.C. (1986). Mechanisms of tumour progression. *Cancer Res.*, **46**, 2203.

POBER, J.S., GIMBRONE, M.A., COTRAN, R.S. & 4 others (1983). An expression by vascular endothelium is inducible by activated T-cells and by human-interferon. *J. Exp. Med.*, **157**, 1339.

POULSEN, L.O., ELLING, P., BRANDT SØRENSEN, F. & HØEDT-RASMUSSEN, K. (1986). HLA-DR expression and disease activity in ulcerative colitis. *Scand. J. Gastroenterol.*, **21**, 364.

RIDELL, R.H., GOLDMAN, H., RANSHOFF, D.F. & 9 others (1983). Dysplasia in inflammatory bowel disease: Standardized classification with provisional clinical applications. *Hum. Pathol.*, **14**, 931.

ROGNUM, T.O., BRANDTZAEG, P., BAKLIEN, K. & HOGNESTAD, J. (1979). Immunoglobulin-producing cells in the 'transitional' mucosa adjacent to adenocarcinomas of the human large bowel. *Int. J. Cancer*, **23**, 165.

ROGNUM, T.O., BRANDTZAEG, P., OERJASAETER, H., ELGJO, K. & HOGNESTAD, J. (1980). Immunohistochemical study of secretory component, secretory IgA and carcinoembryonic antigen in large bowel carcinomas. *Pathol. Res. Pract.*, **170**, 126.

ROGNUM, T.O., ELGJO, K., FAUSA, O. & BRANDTZAEG, P. (1982*a*). Immunohistochemical evaluation of carcinoembryonic antigen, secretory component, and epithelial IgA in ulcerative colitis with dysplasia. *Gut*, **23**, 123.

ROGNUM, T.O., FAUSA, O. & BRANDTZAEG, P. (1982*b*). Immunohistochemical evaluation of carcinoembryonic antigen, secretory component, and epithelial IgA in tubular and villous large bowel adenomas with different grades of dysplasia. *Scand. J. Gastroenterol.*, **17**, 341.

ROGNUM, T.O., ELGJO, K., BRANDTZAEG, P., OERJASAETER, H. & BERGAN, A. (1982*c*). Plasma carcinoembryonic antigen concentrations and immunohistochemical patterns of epithelial marker antigens in patients with large bowel carcinoma. *J. Clin. Pathol.*, **35**, 922.

ROGNUM, T.O., BRANDTZAEG, P. & THORUD, E. (1983). Is heterogeneous expression of HLA-DR antigens and CEA along with DNA-profile variations evidence of phenotypic instability and clonal proliferation in human large bowel carcinomas? *Br. J. Cancer*, **48**, 543.

SELBY, W.S., JANOSSY, G., MASON, D.Y. & JEWEL, D.P. (1983). Expression of HLA-DR antigen by colonic epithelium in inflammatory bowel disease. *Clin. Exp. Immunol.*, **53**, 614.

SHIELDS, H.M., BATES, M.L., GOLDMAN, H. & 6 others (1985). Scanning electron microscopic appearance of cronic ulcerative colitis with and without dysplasia. *Gastroenterology*, **89**, 62.

SOLLID, L.M., KVALE, D., BRANDTZAEG, P., MARKUSSEN, G. & THORSBY, E. (1987). Interferon $\gamma$ enhances expression of secretory component, the epithelial receptor for polymeric immunoglobulins. *J. Immunol.*, **138**, 4303.

STAVE, R. & BRANDTZAEG, P. (1977). Fluorescence staining of gastric mucosa. A study with special reference to parietal cells. *Scand. J. Gastroenterol.*, **12**, 885.

SVENNEVIG, J.-L. (1980). *In situ* identification of inflammatory cells in malignant, non-lymphoid human tumours. *Acta. Pathol. Microbiol. Scand.*, (A)., **88**, 387.

THRANE, P., SOLLID, L.M., BRANDTZAEG, P. & HAANES, H.R. (1987). Increased expression of secretory component (SC) and HLA-DR in salivary gland epithelium from patients with Sjøgren's syndrome. (Submitted).

VALNES, K., BRANDTZAEG, P., ELGJO, K. & STAVE, R. (1984). Specific and nonspecific humoral defence factors in the epithelium of normal and inflamed gastric mucosa. Immunohistochemical localization of immunoglobulins, secretory component, lysozyme, and lactoferrin. *Gastroenterology*, **86**, 402.

VATN, M.H., ELGJO, K. & BERGAN, A. (1984). Distribution of dysplasia in ulcerative colitis. *Scand. J. Gastroenterol.*, **19**, 893.

YARDLEY, J.H. & KEREN, D.F. (1974). 'Precancer' lesions in ulcerative colitis. A retrospective study of rectal biopsy and colectomy specimens. *Cancer*, **34**, 835.